# Very early tilt-table verticalization unmasks impaired cerebral autoregulation within 24 h after endovascular thrombectomy: a transcranial Doppler study

**DOI:** 10.3389/fneur.2025.1709991

**Published:** 2026-01-06

**Authors:** Julian Deseoe, Yannik Rottenberger, Theodor Pipping, Anne Schwarz, Aurelia Lehmann, Janne M. Veerbeek, Susanne Wegener, Andreas R. Luft, Jeremia P. O. Held, Christoph Globas

**Affiliations:** 1 Department of Neurology, University Hospital Zurich and University of Zurich, Zürich, Switzerland; 2 Neuroscience Center Zurich, Zürich, Switzerland; 3 University of California, Los Angeles, Los Angeles, CA, United States; 4 Neurocenter, Luzerner Kantonsspital, Lucerne, Switzerland; 5 Cereneo Center for Neurology and Rehabilitation, Vitznau, Switzerland; 6 Bellevue Medical Group, Zürich, Switzerland

**Keywords:** blood pressure, cerebral autoregulation, Doppler ultrasound, ischemic stroke, very early mobilization

## Abstract

**Introduction:**

Impaired cerebral autoregulation in the acute phase after ischemic stroke may compromise cerebral perfusion during very early mobilization, posing a risk for secondary injury.

**Methods:**

This observational cross-sectional study investigated the mechanistic response of cerebral and systemic hemodynamics during progressive tilt-table verticalization in 25 patients within 24 h post-thrombectomy and 31 healthy age-matched controls. Middle cerebral artery blood flow velocity (CBFV) was continuously recorded via transcranial Doppler across six full-body tilt positions (−5° to 70°), alongside blood pressure, heart rate, and oxygen saturation.

**Results:**

CBFV declined progressively with increasing tilt in both groups (*p* < 0.001), but the reduction at 70° was significantly greater in stroke patients (*p* = 0.007), indicating compromised cerebral pressure-flow regulation. Diastolic blood pressure, linked to systemic vascular resistance, increased with tilt in controls but failed to rise in stroke patients, remaining significantly lower throughout.

**Discussion:**

Our results suggest impairments in cerebral and systemic vascular control mechanisms in acute stroke patients post thrombectomy. These results provide further insight into the physiological constraints limiting early verticalization and underscore the value of real-time CBFV monitoring as a potential biomarker for autoregulatory integrity. Incorporating individualized hemodynamic profiling may enhance safety and precision in early stroke rehabilitation protocols.

**Clinical trial registration:**

ClinicalTrials.gov, identifier NCT04573114.

## 1 Introduction

Stroke is one of the leading causes of death and disability worldwide, with most strokes being ischemic (1). Despite major advances in reperfusion therapy, functional outcomes often remain suboptimal, particularly in patients with large-vessel occlusions. Prolonged immobilization post ischemic stroke can have several adverse effects, from pneumonia to loss in muscle mass, which can impede rehabilitation (2).

Very early mobilization (VEM), has therefore emerged as a strategy to improve post-stroke outcomes (3). VEM is usually defined as start of active out-of-bed physical therapy (or mobilization) within 24 h post stroke and delivered with a higher dosage and frequency than usual care (3, 4). However, the clinical effectiveness of VEM remains debated. No clear evidence exists, that very early rehabilitation improves functional outcome in ischemic stroke patients (5, 6). Moreover, some trials have even suggested potential harm from overly intensive early mobilization protocols (4). A common criticism of the cited studies is that cerebral blood flow (CBF) during mobilization was not measured (7). It is known that cerebral blood flow velocity (CBFV) decreases in healthy adults when moving to upright body positions (8, 9). Hence it is possible, that early mobilization could lead to decrease in cerebral blood flow in ischemic stroke patients, increasing conversion of ischemic penumbra to core, leading to worse functional outcome (10). This is particularly relevant given the known impairment of cerebral autoregulation in the acute post-stroke period, even after successful recanalization (11). Reduced autoregulatory capacity may limit the brain’s ability to maintain stable perfusion during postural stress.

Verticalization, i.e., bringing patients to an upright body position using a robotic tilt table device with a dynamic foot support, has been studied as preliminary step to ambulation training and as a method to deliver standardized mobilization in the subacute post stroke phase (12). It offers a controlled, reproducible, and passive means of mobilization, making it an ideal model to study cerebrovascular responses in the early post-stroke setting.

To better understand cerebral blood flow and systemic hemodynamics during early mobilization after ischemic stroke, we measured cerebral blood flow velocity (CBFV) and hemodynamic parameters in patients undergoing early, progressive verticalization within 24 h after thrombectomy. To contextualize and interpret stroke-specific responses, we compared these data with a reference cohort of healthy, age-matched adults who underwent the identical verticalization protocol (8).

The primary aim was to assess changes in CBFV during verticalization in ischemic stroke patients < 24 h post thrombectomy. We hypothesized that stroke patients would show a more pronounced reduction in CBFV and altered systemic hemodynamic responses during verticalization compared with healthy individuals, which could potentially serve as a marker for adverse response to early mobilization.

## 2 Materials and methods

### 2.1 Setting and participants

This study was a single center cross-sectional study with a prospective observational design. It was conducted at the University Hospital Zurich in accordance with the protocol and the Declaration of Helsinki, and was approved by the cantonal ethics committee of Zurich (BASEC-Nr. 2020–01732). The study was prospectively registered at ClinicalTrials.gov (Identifier: NCT04573114). Owing to its exploratory nature, no formal sample size calculation was performed. We previously published data from 20 healthy participants (8). To achieve improved group-matching with the stroke group, we recruited an additional eleven healthy participants of similar age using identical inclusion and exclusion criteria via flyers and personal contacts. Eligibility criteria for healthy individuals included age ≥18 years and absence of any known neurological, cardiovascular, or systemic condition potentially affecting cerebral autoregulation.

All medications which healthy participants took, were recorded but not paused or altered for the study.

Twenty-eight ischemic stroke patients being treated at the stroke unit of the University Hospital Zurich were recruited. Inclusion criteria were first ever ischemic stroke affecting the vascular territory of the MCA (distal ICA, M1, M2 or M3 segment) proven by CT- or MR- angiography (extra- and intracranial brain supplying arteries) conducted at admission, inclusion ≤ 24 h after mechanical thrombectomy, GCS > 14, hemodynamic stability (blood pressure: MAP ≥ 70 mmHg without need of catecholamines, heart rate 50–120/min) and age > = 18 years. Excluded were subjects for whom the treating physician opted against verticalization attempts (e.g., cardiopulmonary instability) and those with pre-existing diseases affecting CBF (e.g., cerebral arteriovenous malformations, tumors, hyperviscosity syndromes, etc.). Additional exclusion criteria were an insufficient temporal bone window for stable insonation, venous thromboembolism or symptomatic intracranial hemorrhage. Medication administered before measurements was recorded from hospital records. Infarct location, pretreatment National Institute of Health Stroke Scale (NIHSS) Score and success of recanalization quantified through modified Treatment in Cerebral Infarction (mTICI) Score assessed angiographically at the end of the intervention, were extracted from hospital records. Stroke lateralization was used to guide the selection of the insonated hemisphere. Before verticalization, a NIHSS Score was calculated by a qualified member or the study staff. All measurements were performed between 10/2020 and 12/2024 at the Department of Neurology, University Hospital Zurich, Switzerland.

### 2.2 Measurements

We followed the protocol we previously described (7). For verticalization, we used the Sara Combilizer. Cerebral Blood Flow Velocity (CBFV) as a surrogate marker for cerebral blood flow (CBF) was measured using a Holter Transcranial Doppler (TCD) ultrasound over the Middle Cerebral Artery (MCA). In healthy participants, the MCA of the dominant hemisphere was insonated, the affected hemisphere was insonated for stroke patients. We based our method on the method described by Schaafsma et al. (13).

A 1.5 MHz TCD probe (Transcranial Doppler Holter, Atys Medical, France) was employed, insonating the MCA at a depth of 46–54 mm with a gate length of 8–11 mm. Systolic, diastolic, and mean CBFV values were continuously recorded. For analysis, the mean flow velocity per second was extracted. Mean cerebral blood flow velocity (mean CBFV) was derived from the Doppler waveform as the time-averaged maximum velocity over each cardiac cycle (beat-to-beat). These beat-to-beat values were exported and averaged over predefined time windows for each body position. The initial beat-to-beat extraction (Vsyst, Vdiast, mean CBFV) was performed automatically by the manufacturer’s TCD software. Signal quality was monitored in real-time, and brief signal loss episodes (<15 s) due to probe shift were excluded from analysis. Blood pressure (BP) and heart rate (HR) were measured every minute using a wrist monitor (BC 58, Beurer, Germany) on the left arm, ensuring the monitor remained at the level of the heart via an armrest throughout the protocol. Oxygen saturation was continuously measured with a pulse oximeter clip (PO-100, Pulox, Germany) placed on the right index finger and recorded every minute. All monitoring equipment was validated for clinical use and regularly calibrated. Participants were instructed to remain relaxed and limit movement throughout the protocol. Any adverse effects (e.g., dizziness, nausea, discomfort) were recorded, and a physician was present during all sessions.

#### 2.2.1 Verticalization protocol

**Figure 1:**
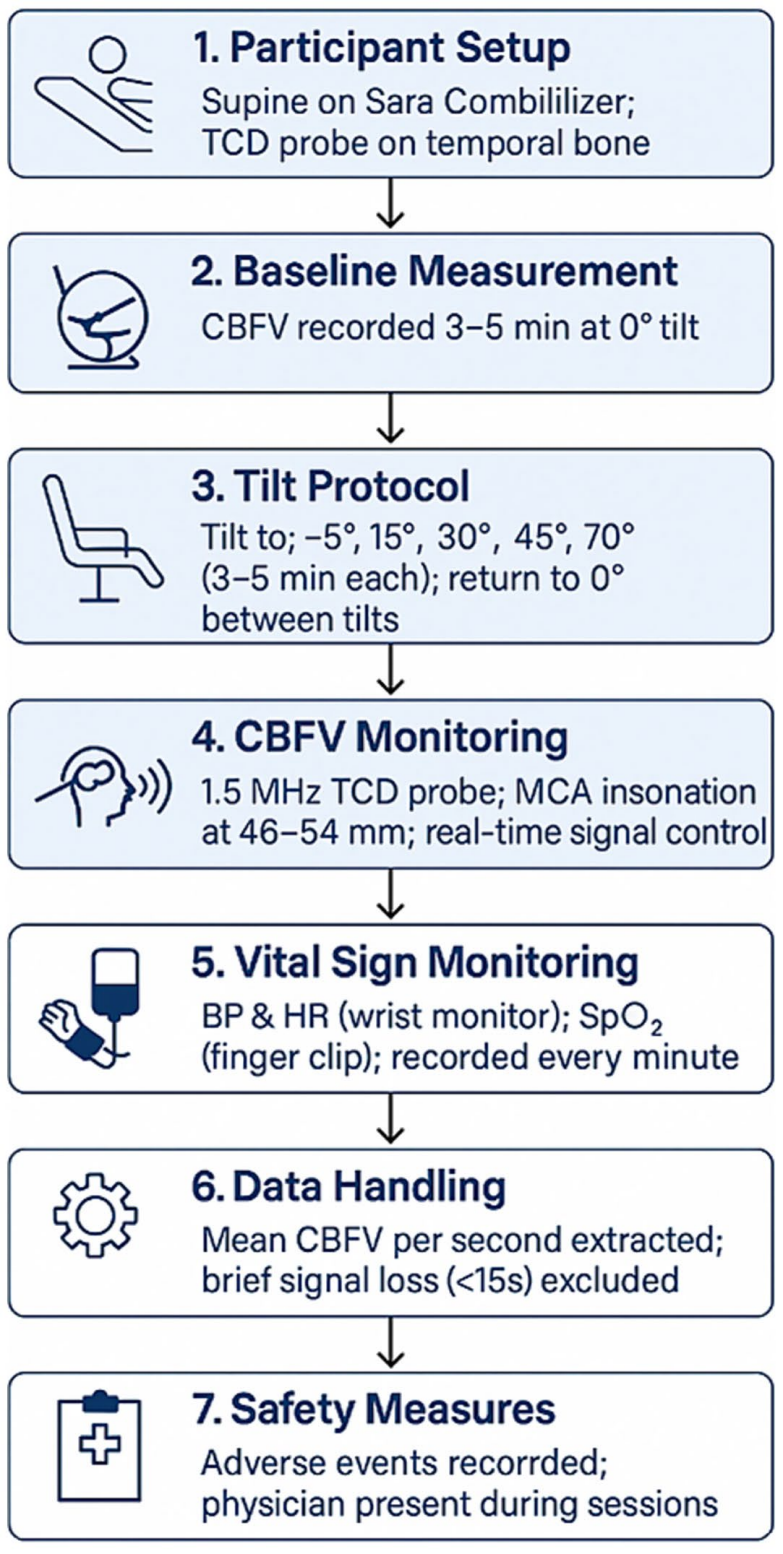
Verticalization protocol. TCD, Transcranial Doppler Device; CBFV, Cerebral blood flow velocity; MCA, middle cerebral artery.

Participants were positioned supine on the Sara Combilizer. A skilled technician placed the Transcranial Doppler (TCD) probe over the temporal bone of the dominant hemisphere for healthy adults and over the affected hemisphere for stroke patients using 1.5 MHz probes secured with an adjustable TCD headframe ensuring stable probe positioning throuought the protocol. Baseline CBFV was recorded for 3–5 min in the 0° tilt (horizontal) position. Subsequently, subjects were tilted to −5° (head-down tilt), 15°, 30°, 45°, and 70° in that order, with measurements taken for 3–5 min at each position. After each tilt, participants were returned to the 0° position for 3–5 min to assess any changes at 0° position over time. The final phase included a return to 0° after the 70° tilt. Position changes were made at a rate of approximately 2° per second. All position changes were passive and assisted by the device, without patient engagement of postural muscles. Participants were instructed to remain as still as possible during measurements to minimize artifacts. The verticalization protocol is shown in Figure 1.

### 2.3 Statistical analysis

All statistical analyses were performed using RStudio software (2024.09.1 + 394) with R version 4.4.2 (14–16). For every position and participant, the mean CBFV, systolic and diastolic BP, HR and oxygen saturation was calculated. The first minute at each position was excluded, to allow circulatory parameters to adjust to the new position. Then, the mean and standard deviation of all participants for each position was calculated for CBFV, BP, HR and oxygen saturation. Baseline was defined as the first 3–5 min at 0° for all parameters. The differences between the phases at 0° were inspected for trends throughout the protocol to detect any potential measurement bias over time. In patients with missing measurements, all available measurements were included in the statistical analysis. No imputation of missing values was done.

Linear mixed-effects models were used to analyze repeated measurements, with verticalization angle included as a fixed effect and participant ID as a random intercept to account for within-subject correlation. Models included additional fixed effects for group (stroke vs. control) and an interaction term (position × group). All models were adjusted for age, sex and usage of antihypertensive drugs. Age and sex have been previously shown to have an effect on CBFV (9).

Sensitivity analyses were conducted to evaluate model robustness: Firstly, by excluding statistical outliers (defined as values with standardized residuals >3 or < −3) and secondly by excluding all participants and patients using antihypertensive medications. We performed an additionial sensitivity analysis by further adjusting our model for body mass index (BMI), as this could affect CBFV (9).

Normality of residuals was assessed using qqplots and for models with outlier residuals, robust linear mixed models were tested as part of the sensitivity analyses described above. Similarly, linear mixed effects models were used to assess the association of position changes with changes in systolic and diastolic BP, HR and oxygen saturation. All *p*-values were two-tailed, and statistical significance was set at *p* < 0.05.

## 3 Results

A total of 31 healthy participants were included in the study. In one participant CBFV measurements at −5° and 15°, as well as heart rate at −5°, could not be recorded for technical reasons. All other measurements were included in the analysis. In two healthy participants, the TCD device lost proper positioning and recorded 0 cm/s of CBFV for durations between 2 and 12 s. These data points were excluded from the analysis. Twenty-eight ischemic stroke patients were enrolled. Two were excluded due to insufficient temporal bone windows, one patient was excluded for withdrawal of consent, leaving 25 patients with recorded measurements. In one patient, measurements were terminated after 45° position due to technical difficulties. In two patients the position of the Doppler probe shifted during the protocol, resulting in implausibly large differences in CBFV between repeated 0° phases (>20 cm/s). Consequently, all data collected after the shift were excluded from analysis. The shift occurred after baseline position in one patient and after 15° in the second. All remaining valid data from these patients were retained for statistical analysis. We did not observe any serious adverse events related to the study. An overview of the characteristics of the healthy participants and stroke patients can be found in Table 1.

**Table 1:** Characteristics of healthy participants and stroke patients.

Parameters	Healthy participants	Stroke patients	*p*-value
*n*	31	25	
Sex (male) (%)	15 (48.4)	15 (60.0)	0.424
Age (mean (SD)) [years]	64.48 (7.95)	66.60 (11.64)	0.551
Antihypertensive medication (%)			
None	22 (71)	18 (72)	
Oral	9 (29.0)	5 (20)	0.230
Intravenous	0 (0)	2 (8)	
Oral antidiabetic medication (%)	0 (0)	2 (8)	0.379
Intravenous thrombolysis (%)		12 (48)	
Time after thrombectomy (mean (SD)) [hours]		19.22 (4.42)	
Pre-thrombectomy NIHSS (median (IQR))		6.50 (2.75, 12.00)	
NIHSS before verticalization (median (IQR))		1.00 (0.00, 3.50)	
mTICI score (%)			
2b		2 (8)	
2c		8 (32)	
3		15 (60)	
Affected hemisphere (left) (%)		8 (32)	
Affected artery (%)			
MCA M1		12 (48)	
MCA M2		11 (44)	
MCA M3		1 (4)	
ICA		1 (4)	

*p*-values were calculated using unpaired *t*-test for continuous variables and chi-squared test for categorial variables. SD, standard deviation; IQR, interquartile range; NIHSS, National institute of health stroke score; mTICI, modified treatment in cerebral infarction.

The healthy and stroke cohorts were reasonably well matched in terms of age (64.5 (SD 7.95) vs. 66.6 (11.6); *p* = 0.55) and sex (male: 48.4% vs. 60%; *p* = 0.42) (Table 1). Nevertheless, all analyses were adjusted for age and sex to account for any residual bias. The use of oral antihypertensive medication was present in both groups. Two patients received intravenous antihypertensive medication shortly before verticalization. Given the medication’s duration of action, the hemodynamic response during verticalization may have been influenced in these participants.

All stroke patients, except one, demonstrated occlusion of the MCA, most commonly involving M1 and M2 segments in CT- or MR-angiography conducted before thrombectomy. None of the included stroke patients had additional proximal carotid stenosis or occlusion. 12 stroke patients (48%) additionally received intravenous thrombolysis as bridging therapy before thrombectomy. All included stroke patients demonstrated at least substantial reperfusion of the affected vessel following thrombectomy, defined as mTICI ≥2b, with the majority achieving near-complete or complete reperfusion (mTICI 2c-3). Stroke severity prior to thrombectomy varied, as reflected by a wide range of pre-treatment NIHSS scores. (median: 6.50, IQR 2.75–12.00) Following thrombectomy and prior to verticalization, most patients exhibited reduced neurological deficits (NIH-SS at admission: median 6.5, IQR 2.75–12, NIH-SS before verticalization: median: 1.0, IQR: 0–3.50) (Table 1). No severe adverse events of verticalization were observed.

The mean and standard deviation of CBFV, systolic BP, diastolic BP, HR and oxygen saturation are presented in Table 2. As an initial step, all 0° phases were evaluated for potential systematic changes over the course of the protocol. As shown in Table 2, all parameters remained stable across the repeated 0° phases, suggesting no protocol drift or cumulative effect of time. For further analysis only the phases with increasing levels of verticalization were assessed.

**Table 2:** Mean and standard deviation of cerebral blood flow velocity (CBFV), systolic blood pressure (BP), diastolic blood pressure (BP), heart rate (HR) and oxygen saturation (SpO2) at different phases of verticalization protocol.

Position	CBFV	Systolic BP	Diastolic BP	HR	SpO2
Healthy participants (*n* = 31)					
Baseline	44.7 (11.7)	134.7 (12.0)	77.2 (7.7)	68.0 (9.7)	95.7 (2.4)
−5°	45.5 (11.7)	128.5 (11.8)	74.0 (7.9)	66.7 (9.6)	95.4 (2.3)
0° (2)	44.7 (11.9)	130.5 (10.5)	75.1 (6.8)	65.9 (9.7)	95.4 (2.2)
15°	44.0 (11.5)	133.9 (13.6)	77.8 (6.6)	66.0 (9.8)	95.4 (2.4)
0° (3)	45.1 (11.3)	131.2 (12.9)	74.9 (7.3)	65.3 (9.7)	95.1 (2.4)
30°	42.3 (10.7)	132.1 (12.0)	82.0 (7.5)	67.9 (9.3)	95.5 (2.0)
0° (4)	44.4 (11.6)	130.5 (11.8)	74.9 (5.6)	63.4 (9.3)	95.0 (2.4)
45°	40.1 (10.9)	133.7 (11.6)	83.0 (7.5)	70.6 (9.6)	95.3 (2.5)
0° (5)	44.2 (11.1)	130.0 (11.7)	75.1 (6.7)	63.2 (9.0)	94.9 (2.4)
70°	38.5 (10.9)	133.8 (15.7)	82.2 (8.0)	76.2 (10.1)	95.6 (2.1)
0° (6)	43.9 (17.8)	133.8 (13.3)	76.0 (6.7)	63.4 (9.6)	95.2 (2.5)
Stroke patients (*n* = 25)					
Baseline	51.5 (17.8)	128.7 (22.3)	62.1 (16.0)	68.1 (16.1)	96.0 (1.9)
−5°	51.3 (18.5)	130.5 (21.4)	61.7 (15.2)	68.6 (15.3)	95.7 (2.2)
0° (2)	51.5 (18.7)	130.6 (20.3)	62.1 (14.4)	68.4 (15.5)	95.9 (2.1)
15°	50.4 (18.4)	129.4 (19.9)	60.8 (13.2)	68.5 (15.3)	95.9 (2.3)
0° (3)	52.2 (18.6)	130.8 (20.1)	60.6 (15.0)	67.8 (15.5)	95.8 (2.4)
30°	49.1 (18.0)	128.4 (21.3)	59.7 (13.4)	69.0 (17.2)	96.0 (2.3)
0° (4)	50.9 (18.3)	131.1 (20.6)	60.7 (14.8)	66.6 (16.4)	96.1 (2.2)
45°	46.0 (16.0)	125.9 (21.7)	61.0 (13.4)	73.4 (19.0)	96.6 (2.2)
0° (5)	51.3 (17.7)	130.5 (21.1)	61.0 (15.2)	66.0 (16.7)	95.7 (2.2)
70°	41.1 (16.9)	122.7 (25.1)	59.9 (13.9)	78.0 (20.2)	96.8 (2.1)
0° (6)	53.2 (20.6)	131.1 (22.5)	62.3 (15.2)	65.4 (16.1)	95.7 (2.0)

**Figure 2:**
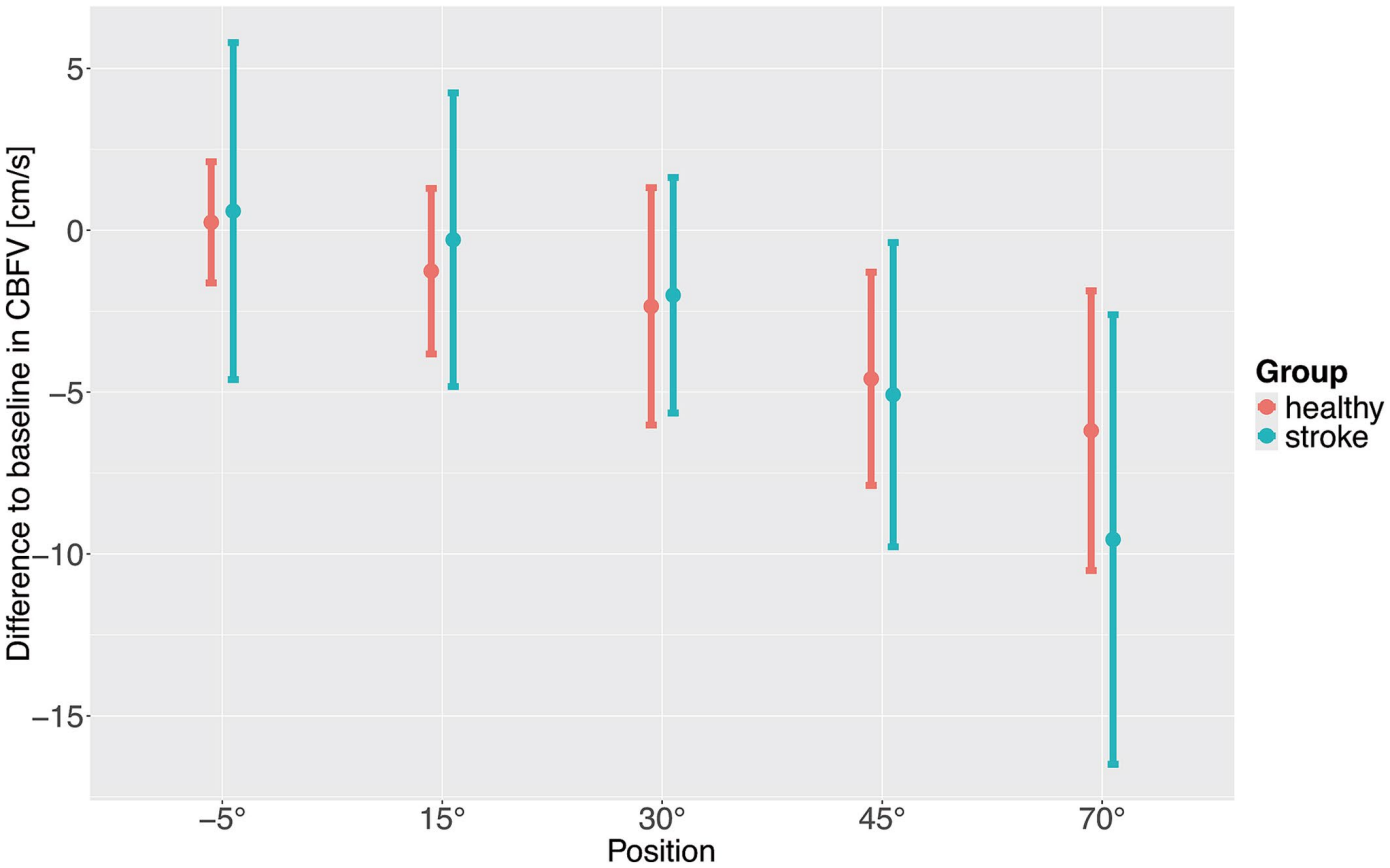
Difference to baseline of cerebral blood flow velocity (CBFV) with increasing levels of verticalization positions. Points show means, error bars show standard deviation.

To investigate the changes in hemodynamic parameters during increasing levels of verticalization linear mixed effects models were employed. CBFV dropped significantly with increasing levels of verticalization in both healthy participants and stroke patients (Table 3; Figure 2). The drop was significant starting at 30°. Stroke patients had a higher CBFV at baseline, although this difference was not statistically significant. CBFV dropped significantly stronger in stroke patients compared to healthy patients at 70° of verticalization. Age and sex were not associated with a significant difference in CBFV. Usage of oral as well as intravenous antihypertensive drugs were associated with lower CBFV. This was significant only for oral antihypertensive drugs, although our sample size for patients receiving intravenous antihypertensive drugs was very small. To assess a potential influence of antihypertensive medications, a sensitivity analysis was conducted, removing all participants and patients receiving antihypertensive drugs. This did not impact the results (Supplementary Table S1). In the analysis of residuals, four potential outliers were identified (Supplementary Figure S1). Removing these outliers did not modify the results (Supplementary Table S2). Further adjusting the model for BMI did also not modify the results. (Supplementary Table S3).

**Table 3:** Linear mixed effects model.

Parameters	Estimate	Std. Error	95% CI (min/max)	*p*-value
(Intercept)	38.5	12.8	13.4 / 63.6	0.004
−5°	0.3	0.8	−1.4 / 1.9	0.76
15°	−1.2	0.8	−2.9 / 0.4	0.14
30°	−2.4	0.8	−4.0 / −0.7	**0.005**
45°	−4.6	0.8	−6.2 / −3.0	**< 0.001**
70°	−6.2	0.8	−7.8 / −4.6	**< 0.001**
Age [y]	0.2	0.2	−0.2 / 0.6	0.41
Sex (male)	−3.0	3.9	−10.5 / 4.6	0.44
Antihypertensives (oral)	−10.6	4.4	−19.2 / −1.9	**0.02**
Antihypertensives (intravenous)	−6.7	10.5	−27.3 / 13.9	0.52
Group (stroke)	6.4	4.0	−1.4 / 14.2	0.11
Difference at −5°	0.3	1.3	−2.2 / 2.8	0.81
Difference at 15°	0.9	1.3	−1.6 / 3.4	0.47
Difference at 30°	0.4	1.3	−2.1 / 2.9	0.75
Difference at 45°	−0.4	1.3	−2.9/2.1	0.74
Difference at 70°	−3.5	1.3	−6.0 / −1.0	**0.007**
*N* = 56, observations = 325				

CBFV measured in cm/s was the independent variable. Position was encoded as five binary variables with baseline as reference. Overall *p* value for the impact of position was *p* < 0.001. Sex was encoded as binary variable with female as reference category. Antihypertensives were encoded as two binary variables (oral and intravenous) with no antihypertensives as reference category. Group is a binary variable encoding whether patients were healthy participants or stroke patients. Healthy participants were taken as reference. An interaction between group and position was included in the model. Results are shown under “Difference at” with the corresponding position. Here healthy participants are also the reference category. The individual test subjects were included as random intercepts. *p*-values were calculated using Satterwhaite’s method. Bold values indicate significant *p*-values (*p* < 0.05).

**Figure 3:**
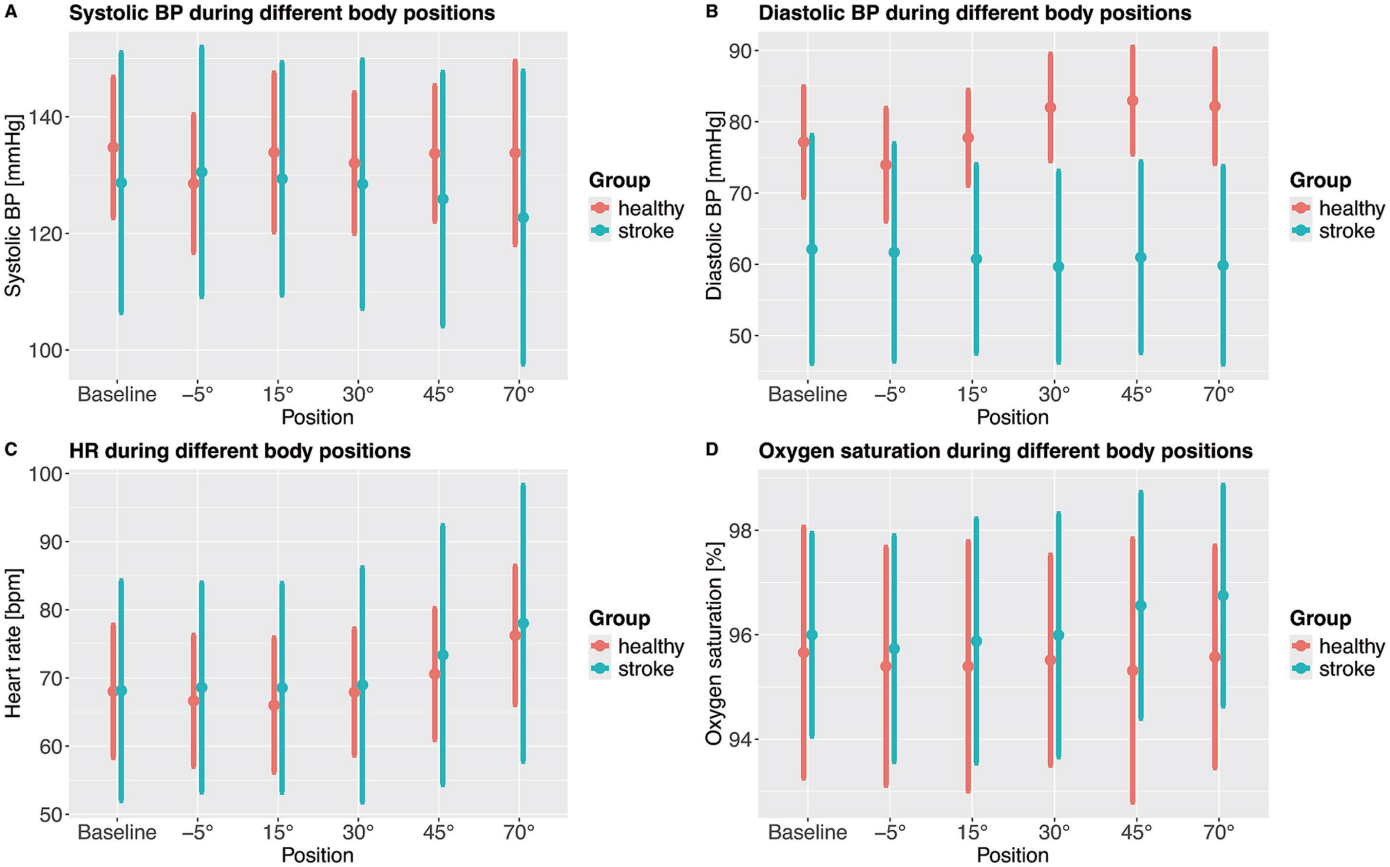
Mean systolic blood pressure **(A)**, diastolic blood pressure **(B)**, heart rate **(C)** and oxygenation **(D)** during different verticalization positions. Points show means and error bars show standard deviation. BP, blood pressure; HR, heart rate.

In healthy subjects, systolic BP dropped significantly at −5° (head down). This drop was not observed in stroke patients (Figure 3), multivariate models adjusted for age, sex and hypertensive use are shown in Supplementary Tables S4–S7. Apart from that, there were no significant changes in systolic BP and no significant difference between healthy participants and stroke patients during verticalization.

Stroke patients demonstrated lower diastolic blood pressure for all levels of verticalization compared to healthy adults. In addition, while it significantly increased with increasing levels of verticalization in healthy participants, starting at 30° of head up tilt, it stayed constant in stroke patients. A sensitivity analysis was conducted, by removing all participants and patients using antihypertensive drugs, which did not change the results (Supplementary Table S8). To further understand changes in BP, we investigated how many patients and healthy participants met criteria for orthostatic hypotension from 0° to 70° tilt. Orthostatic hypotension was defined as either >20 mmHg drop in systolic BP or >10 mmHg drop in diastolic BP (17). 3 out of 24 assessed patients (12.5%) patients showed orthostatic hypotension with drop in BP from baseline to 70° head-up tilt. One healthy participant out of 31 (3.2%) showed orthostatic hypotension. None of these participants experienced serious adverse events.

HR increased significantly with increasing levels of verticalization in healthy participants and stroke patients, with no significant differences between the two groups.

SpO2 increased in stroke patients compared to baseline at 45° and 70° of head up tilt, while it remained constant in healthy participants, leading to statistically significant effects in the linear mixed effects model. Because the difference was less than one percentage point, it may lack clinical relevance. Overall, there was more variability in hemodynamic parameters in stroke patients compared to healthy adults as evidenced by larger standard deviations. An overview over hemodynamic parameters during verticalization can be found in Figure 3.

**Figure 4:**
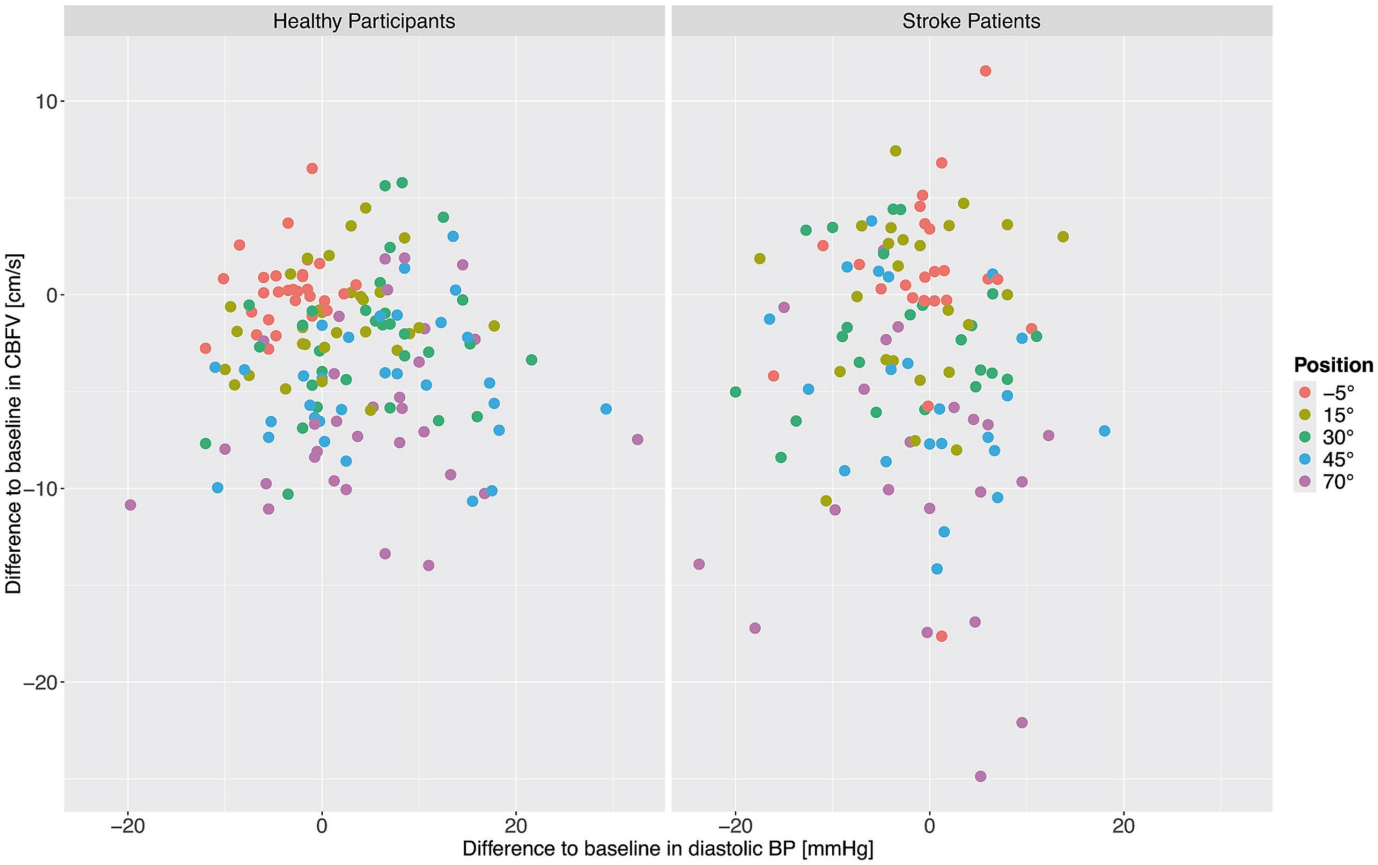
Change from baseline in cerebral blood flow velocity (CBFV) and diastolic blood pressure (BP) for healthy adults (left) and stroke patients (right). Each point shows the mean CBFV and diastolic BP for one patient or participant at one position. The different positions are represented by different colors.

Finally, it was investigated whether changes in CBFV were associated with the changes in diastolic BP during verticalization. We assessed correlations between change in CBFV from baseline and change in diastolic BP from baseline during verticalization for stroke patients and for healthy participants using Pearsons’s correlation coefficient. No significant correlations were found for healthy participants (*r* = −0.09, 95% CI −0.23–0.06, *p* = 0.25) and stroke patients (*r* = −0.02, 95% CI −0.18–0.15, *p* = 0.82). A graphical overview can be found in Figure 4, which shows mean CBFV and diastolic BP per position per patient and participant.

## 4 Discussion

The aim of the study was to assess changes in CBFV and hemodynamic parameters during very early verticalization (< 24 h) post thrombectomy in comparison to healthy controls. In summary, CBFV decreased with increasing levels of verticalization in both healthy adults and stroke patients. CBFV dropped significantly stronger in stroke patients compared to healthy adults at 70° of head up tilt, indicating impaired CBF regulation. Systolic BP remained rather constant in both healthy participants and stroke patients. Diastolic BP increased with increasing levels of verticalization in healthy participants while it remained constant in stroke patients. Overall diastolic BP was lower in stroke patients than in healthy participants. HR increased significantly in both stroke patients and healthy adults, with no significant differences between the groups. SpO2 remained constant during verticalization in both groups. The differences in CBFV and diastolic BP between the two groups persisted in sensitivity analyses excluding individuals receiving antihypertensive medications. We found no evidence of changes in diastolic BP during verticalization being associated with changes in CBFV.

CBFV and hemodynamic responses to verticalization in healthy adults were consistent with previous reports (7, 9). For stroke subjects, CBFV has been shown to decline during verticalization at time points greater than 24 h after stroke. In this study, only the minority of patients had large vessel occlusion (18). Similar results were obtained for raising the head (19). To our knowledge, our study is the first to demonstrate a CBFV decline during verticalization in the high risk group with large-vessel occlusion at admission and verticalized during the hyperacute stage < 24 h post-thrombectomy. These effects were observed despite the fact that the majority of patients were recanalized successfully (mTICI Score: 2c-3 in 92%).

There is increasing evidence that blood pressure regulation is disrupted after stroke, including high rates of orthostatic hypotension (20–22). Carvalho et al. who measured diastolic BP during mobilization 48 h post stroke, found smaller increase in diastolic BP in stroke patients compared to healthy adults, although this was not statistically significant (18).

We interpret the stronger CBFV decline in stroke patients as evidence of impaired dynamic cerebral autoregulation. It is important to note that changes in CBFV are only representative for changes in cerebral blood flow (CBF) if the diameter of the vessel does not change. It has been shown that this is the case for the MCA at least during simulated orthostasis (23). Therefore, changes in CBFV can be assumed to represent CBF during orthostasis. CBFV falls during verticalization because cerebral perfusion pressure decreases together with arterial cerebral pressure due to the drop in hydrostatic pressure in the upright position (24). In the healthy population, cerebral autoregulation aims to counteract this drop in cerebral perfusion pressure, trying to keep CBF and CBFV constant by reducing cerebral vascular resistance (25). It has been shown that patients after stroke show impairment of CBF regulation with failure to adequately respond to changes in cerebral perfusion pressure (26) Proposed mechanisms include arteriolar vasospasm, reduced vasodilator activity and diminished metabolic demand in necrotic tissue. There is also increasing evidence that despite complete recanalization, stroke patients can show incomplete reperfusion in the affected territory (“no reflow phenomenon”) (27). We hypothesize that, as a result of these mechanisms, stroke patients are unable to sufficiently lower cerebrovascular resistance during verticalization, leading to a disproportionate decline in CBF and CBFV.

We hypothesize that the differences in diastolic BP between ischemic stroke patients and healthy participants are due to impairments in regulation of peripheral vascular resistance, as it has been shown that diastolic BP is strongly related to systemic vascular resistance (28). Studies in both humans and mice have suggested that ischemic stroke can lead to defictis in regulation of peripheral vascular resistance (29, 30). The frequency of orthostatic hypotension (defined as ≥20 mmHg systolic or ≥10 mmHg diastolic drop) was higher in the stroke cohort (12.5%) as compared to healthy (3.2%). The fact that CBFV and diastolic BP changes were not correlated points toward distinct, parallel impairments in cerebral and systemic vascular control.

Previous trials on VEM have failed to show an average benefit of very early mobilization regarding favorable functional outcome (mRS 0–2) (4, 5). Patients with strong decrease of CBFV during VEM, may not benefit or even worsen due to hypoperfusion (10). CBFV monitoring could help to identify such patients and may be a method for better patient stratification. Importantly impaired cerebral autoregulation has been linked to worse functional outcomes (31).

Similarly, impaired BP regulation, particularly orthostatic hypotension has been linked to recurrent stroke (32). Early detection of BP dysregulation may support risk stratification and prevention strategies. Our verticalization protocol may be used to identify patients at risk.

One limitation of this study is the lack of controlling for antihypertensive drugs that may have influenced vascular responses. We tried to address this problem by removing all patients and participants taking antihypertensive drugs from the analysis, which did not change results. Another uncontrolled factor is propofol anesthesia which stroke patients received during thrombectomy. With patients undergoing verticalization as early as 10.4 h post thrombectomy, there might still be an effect of the anesthesia on hemodynamic parameters. We consider this influence minimal given the short half-life of propofol between 1 and 3 h and the minor effects on hemodynamic parameters following short interventions (33, 34).

Another disadvantage is our standardized verticalization protocol which may lack real world generalization. The Sara Combilizer chair allowed for reduction of head movements, which is important, as Holter TCD measurements show artifacts when the device moves. Head fixation is usually not fully controlled during conventional mobilization and may aggravate the CBF drops during verticalization. The results may therefore overestimate the effects of conventional mobilization on CBF.

Further, end-tidal CO_2_ was not measured during verticalization. Thus we could not study if the verticalization induced hyperventilation in the participants and patients, a factor that may contribute to cerebral vasoconstriction and reduced CBFV (35). In addition, individual sensitivity to postural changes cold have affected the results. Finally, it is important to note that we do not directly assess cerebral autoregulation. This could be done in future studies using dedicated techniques such as transfer function analysis (36).

In conclusion, ischemic stroke patients post-thrombectomy exhibited a significantly greater decline in CBFV and impaired diastolic BP response during early verticalization compared to healthy adults. Patients also showed significantly lower diastolic BP compared to healthy participants which failed to increase during verticalization. These findings indicate early dysfunction in cerebral autoregulation and systemic vascular control. Measuring CBFV and BP responses during VEM may help predict functional outcomes and guide individualized post-stroke rehabilitation strategies. Future studies should explore the prognostic value of verticalization-induced hemodynamic responses, including CBFV and orthostatic BP changes, to optimize personalized stroke rehabilitation.

## Data Availability

The original contributions presented in the study are included in the article/Supplementary material, further inquiries can be directed to the corresponding author.
