# Supplementary material for: Very early tilt-table verticalization unmasks impaired cerebral autoregulation within 24 h after endovascular thrombectomy: a transcranial Doppler study
Source: Front Neurol. 2026 Jan 6;16:1709991. doi: 10.3389/fneur.2025.1709991 (PMC12815858; doi:10.3389/fneur.2025.1709991)
Supplement: Supplementary file 1 [file Data_Sheet_1.pdf]

Supplementary Material

1. Supplementary Tables

	Estimate	95 % CI (min/max)	P-value
**(Intercept)**	36.4	8.4 / 64.4	0.02
**- 5°**	0.1	-1.0/2.1	0.96
**15°**	-1.4	-3.4 / 0.6	0.18
**30°**	-2.1	-4.1 / 0.0	**0.05**
**45°**	-4.4	-6.4 / -2.4	**< 0.001**
**70°**	-5.7	-7.8/-3.7	**< 0.001**
**Age [y]**	0.2	-0.3/0.6	0.43
**Sex (male)**	-1.7	-10.7/7.2	0.71
**Group (stroke)**	8.4	-0.9/17.6	0.08
**Difference at -5°**	-0.4	-3.5/2.6	0.78
**Difference at 15°**	1.7	-1.3/4.8	0.27
**Difference at 30°**	0.2	-2.8/3.3	0.87
**Difference at 45°**	-1.1	-4.2/1.9	0.46
**Difference at 70°**	-5.4	-8.4/-2.3	**< 0.001**
**N = 40, observations = 234**			

Table S1. *Linear mixed effects model of cerebral blood flow velocity (CBFV) during verticalization. All participants and patients taking antihypertensive drugs were removed. CBFV measured in cm/s was the independent variable. Position was encoded as five binary variables with baseline as reference. Sex was encoded as binary variable with female as reference category. Group is a binary variable encoding whether patients were healthy participants or stroke patients. Healthy participants were taken as reference. An interaction between group and position was included in the model. Results are shown under „Difference at" with the corresponding position. Here healthy participants are also the reference category. The individual test subjects were included as random intercepts. P - values were calculated using Satterwhaite's method.*

	Estimate	95% CI (min / max)	P-value
**(Intercept)**	38.1	12.9 / 63.2	0.005
**- 5°**	0.3	-1.1 / 1.6	0.72
**15°**	-1.2	-2.6 / 0.1	**0.08**
**30°**	-2.4	-3.7 / -1.0	**< 0.001**
**45°**	-4.6	-6.0 / -3.2	**< 0.001**
**70°**	-6.2	-7.6 / -4.8	**< 0.001**
**Age [y]**	0.2	-0.2 / 0.6	0.38
**Sex (male)**	-3.2	-10.8 / 4.4	0.42
**Antihypertensives (oral)**	-10.9	-19.6 / -2.2	**0.02**
**Antihypertensives (intravenous)**	-7.2	-27.9 / 13.5	0.50
**Group (stroke)**	6.4	-1.3 / 14.2	0.11
**Difference at -5°**	0.8	-1.3 / 2.9	0.45
**Difference at 15°**	0.9	-1.2 / 3.0	0.38
**Difference at 30°**	0.4	-1.7 / 2.5	0.70
**Difference at 45°**	-0.4	-2.5 / 1.7	0.69
**Difference at 70°**	-2.3	-4.5 / -0.2	**0.04**
**N = 56, observations = 321**			

Table S2. *Linear mixed effects model for cerebral blood flow velocity (CBFV) during verticalization. Four outliers removed. CBFV measured in cm/s was the independent variable. Position was encoded as five binary variables with baseline as reference. Sex was encoded as binary variable with female as reference category. Antihypertensives were encoded as two binary variables (oral and intravenous) with no antihypertensives as reference category. Group is a binary variable encoding whether patients were healthy participants or stroke patients. Healthy participants were taken as reference. An interaction between group and position was included in the model. Results are shown under „Difference at" with the corresponding position. Here healthy participants are also the reference category. The individual test subjects were included as random intercepts. P - values were calculated using Satterwhaite's method.*

	**Estimate**	**95% CI (min / max)**	**P-value**
**(Intercept)**	45.2	4.2 / 86.2	0.04
**- 5°**	0.3	-1.3 / 1.8	0.74
**15°**	-1.2	-2.8 / 0.3	0.11
**30°**	-2.4	-3.9 / -0.8	**0.002**
**45°**	-4.6	-6.1 / -3.1	**< 0.001**
**70°**	-6.2	-7.7 / -4.7	**< 0.001**
**Group (stroke)**	6.9	-1.7 / 15.4	0.12
**Age [y]**	0.2	-0.3 / 0.6	0.46
**Sex (male)**	-3.4	-11.7 / 4.8	0.42
**Antihypertensives (oral)**	-9.5	-19.3 / 0.3	0.06
**Antihypertensives (intravenous)**	-7.4	-29.1 / 14.3	0.51
**BMI [kg/m^2]**	-0.3	-1.3 / 0.8	0.62
**Difference at -5°**	0.7	-1.7 / 3.0	0.59
**Difference at 15°**	0.3	-2.1 / 2.6	0.82
**Difference at 30°**	-0.0	-2.4 / 2.4	0.99
**Difference at 45°**	-0.7	-3.1 / 1.6	0.54
**Difference at 70°**	-3.9	-6.3 / -1.5	**0.002**
**N = 53, observations = 307**			

Table S3. *Linear mixed effects model for cerebral blood flow velocity (CBFV) during verticalization. Further adjusted for Body mass index (BMI). Patients in which were transferred to another hospital before BMI could be measured were excluded. CBFV measured in cm/s was the independent variable. Position was encoded as five binary variables with baseline as reference. Sex was encoded as binary variable with female as reference category. Antihypertensives were encoded as two binary variables (oral and intravenous) with no antihypertensives as reference category. Group is a binary variable encoding whether patients were healthy participants or stroke patients. Healthy participants were taken as reference. An interaction between group and position was included in the model. Results are shown under „Difference at" with the corresponding position. Here healthy participants are also the reference category. The individual test subjects were included as random intercepts. P - values were calculated using Satterwhaite's method.*

	Estimate	95 % CI	P-value
**(Intercept)**	96.0	13.9 / 68.7	< 0.001
**- 5°**	-6.2	2.0 / -10.2	**0.003**
**15°**	-0.8	2.0 / -4.8	0.68
**30°**	-2.7	2.0 / -6.7	0.19
**45°**	-1.0	2.0 / -5.0	0.62
**70°**	-0.9	2.0 / -4.9	0.65
**Age [y]**	0.7	0.2 / 1.2	**0.004**
**Sex (male)**	-3.5	4.2 / -11.7	0.40
**Antihypertensives (oral)**	-5.3	4.8 / -14.7	0.27
**Antihypertensives (intravenous)**	11.5	11.4 / -10.8	0.32
**Group (stroke)**	-8.4	4.7 / -17.5	0.08
**Difference at -5°**	8.0	3.1 / 2.1	**0.009**
**Difference at 15°**	1.5	3.1 / -4.5	0.62
**Difference at 30°**	2.4	3.1 / -3.6	0.43
**Difference at 45°**	-1.8	3.1 / -7.8	0.56
**Difference at 70°**	-4.2	3.1 / -10.2	0.17
**N = 56, obs = 335**			

Table S4. *Linear mixed effects model for systolic blood pressure during verticalization. Systolic BP measured in mmHg was the independent variable. Position was encoded as five binary variables with baseline as reference. Sex was encoded as binary variable with female as reference category. Antihypertensives were encoded as two binary variables (oral and intravenous) with no antihypertensives as reference category. Group is a binary variable encoding whether patients were healthy participants or stroke patients. Healthy participants were taken as reference. An interaction between group and position was included in the model. Results are shown under „Difference at" with the corresponding position. Here healthy participants are also the reference category. The individual test subjects were included as random intercepts. P - values were calculated using Satterwhaite's method.*

	Estimate	95% CI	P-value
**(Intercept)**	86.6	68.3 / 105.0	< 0.001
**- 5°**	-3.2	-5.8 / -0.6	**0.02**
**15°**	0.6	-2.0 / 3.2	0.65
**30°**	4.9	2.2 / 7.5	**< 0.001**
**45°**	5.8	3.2 / 8.4	**<0.001**
**70°**	5.0	2.4 / 7.6	**<0.001**
**Age [y]**	-0.1	-0.4 / 0.1	0.33
**Sex (male)**	1.2	-4.3 / 6.8	0.66
**Antihypertensives (oral)**	-3.4	-9.7 / 2.9	0.30
**Antihypertensives (intravenous)**	-1.7	-16.7 / 13.3	0.83
**Group (stroke)**	-15.1	-21.2 / -9.0	**< 0.001**
**Difference at -5°**	2.7	-1.2 / 6.7	0.17
**Difference at 15°**	-1.9	-5.8 / 2.0	0.34
**Difference at 30°**	-7.3	-11.2 / -3.4	**<0.001**
**Difference at 45°**	-6.9	-10.8 / -3.0	**<0.001**
**Difference at 70°**	-6.5	-10.5 / -2.6	**0.001**
**N = 56 obs = 335**			

Table S5. *Linear mixed effects model for diastolic blood pressure during verticalization. Diastolic BP measured in mmHg was the independent variable. Position was encoded as five binary variables with baseline as reference. Sex was encoded as binary variable with female as reference category. Antihypertensives were encoded as two binary variables (oral and intravenous) with no antihypertensives as reference category. Group is a binary variable encoding whether patients were healthy participants or stroke patients. Healthy participants were taken as reference. An interaction between group and position was included in the model. Results are shown under „Difference at" with the corresponding position. Here healthy participants are also the reference category. The individual test subjects were included as random intercepts. P - values were calculated using Satterwhaite's method.*

	Estimate	95% CI	P-value
**(Intercept)**	80.4	56.1 / 104.7	3.88E-08
**-5°**	-1.5	-3.5 / 0.5	0.15
**15°**	-2.0	-4.0 / -0.1	**0.05**
**30°**	-0.1	-2.1 / 1.9	0.9146
**45°**	2.5	0.6 / 4.5	**0.01**
**70°**	8.2	6.2 / 10.2	**< 0.001**
**Age**	-0.2	-0.6 / 0.2	0.29
**Gendermale**	0.0	-7.3 / 7.4	0.99
**Antihypertensivesoral**	2.7	-5.7 / 11.1	0.53
**Antihypertensivesinfusion**	-3.1	-23.0 / 16.9	0.77
**Group (stroke)**	1.0	-6.6 / 8.7	0.79
**Difference at -5°**	1.9	-1.0 / 4.9	0.20
**Difference at 15°**	2.4	-0.5 / 5.4	0.11
**Difference at 30°**	0.9	-2.0 / 3.9	0.53
**Difference at 45°**	2.7	-0.3 / 5.6	0.08
**Difference at 70°**	1.8	-1.2 / 4.7	0.24
**N = 56, obs = 334**			

Table S6. *Linear mixed effects model for heart rate during verticalization. Heart rate measured in beats per minute was the independent variable. Position was encoded as five binary variables with baseline as reference. Sex was encoded as binary variable with female as reference category. Antihypertensives were encoded as two binary variables (oral and intravenous) with no antihypertensives as reference category. Group is a binary variable encoding whether patients were healthy participants or stroke patients. Healthy participants were taken as reference. An interaction between group and position was included in the model. Results are shown under „Difference at" with the corresponding position. Here healthy participants are also the reference category. The individual test subjects were included as random intercepts. P - values were calculated using Satterwhaite's method.*

	Estimate	95%CI	P-value
**(Intercept)**	97.4	93.6 / 101.2	< 0.001
**- 5°**	-0.3	-0.7 / 0.2	0.24
**15°**	-0.3	-0.7 / 0.2	0.24
**30°**	-0.1	-0.6 / 0.3	0.51
**45°**	-0.3	-0.8 / 0.1	0.12
**70°**	-0.1	-0.5 / 0.4	0.71
**Age [y]**	-0.03	-0.1 / 0.0	0.37
**Sex (male)**	-0.1	-1.2 / 1.1	0.93
**Group (stroke)**	0.4	-0.8 / 1.6	0.51
**Difference at -5°**	-0.0	-0.7 / 0.6	0.99
**Difference at 15°**	0.1	-0.5 / 0.8	0.67
**Difference at 30°**	0.1	-0.5 / 0.8	0.69
**Difference at at 45°**	0.9	0.3 / 1.6	**0.007**
**Difference at 70°**	0.8	0.2 / 1.5	**0.02**
**N = 56, obs = 335**			

Table S7. *Linear mixed effects model for oxygen saturation during verticalization. Oxygen saturation measured in percent was the independent variable. Position was encoded as five binary variables with baseline as reference. Sex was encoded as binary variable with female as reference category. Antihypertensives were encoded as two binary variables (oral and intravenous) with no antihypertensives as reference category. Group is a binary variable encoding whether patients were healthy participants or stroke patients. Healthy participants were taken as reference. An interaction between group and position was included in the model. Results are shown under „Difference at" with the corresponding position. Here healthy participants are also the reference category. The individual test subjects were included as random intercepts. P - values were calculated using Satterwhaite's method.*

	Estimate	95% CI	P-value
**(Intercept)**	81.3	61.3 / 101.3	< 0.001
**- 5°**	-3.4	-6.4 / -0.4	**0.03**
**15°**	1.8	-1.2 / 4.8	0.23
**30°**	6.5	3.5 / 9.5	**< 0.001**
**45°**	7.6	4.6 / 10.6	**< 0.001**
**70°**	5.7	2.7 / 8.7	**< 0.001**
**Age [y]**	-0.1	-0.4 / 0.2	0.64
**Sex (male)**	0.1	-6.3 / 6.5	0.98
**Group (stroke)**	-12.9	-19.9 / -5.8	**< 0.001**
**Difference at -5°**	2.6	-1.9 / 7.0	0.26
**Difference at 15°**	-2.6	-7.1 / 1.8	0.25
**Difference at 30°**	-8.1	-12.5 / -3.6	**< 0.001**
**Difference at at 45°**	-7.9	-12.3 / -3.4	**< 0.001**
**Difference at 70°**	-7.8	-12.3 / -3.3	**< 0.001**
**N = 40, obs = 239**			

Table S8. *Linear mixed effects model for diastolic blood pressure during verticalization, all patients and participants with antihypertensive medications removed. Diastolic BP measured in mmHg was the independent variable. Position was encoded as five binary variables with baseline as reference. Sex was encoded as binary variable with female as reference category. Antihypertensives were encoded as two binary variables (oral and intravenous) with no antihypertensives as reference category. Group is a binary variable encoding whether patients were healthy participants or stroke patients. Healthy participants were taken as reference. An interaction between group and position was included in the model. Results are shown under „Difference at" with the corresponding position. Here healthy participants are also the reference category. The individual test subjects were included as random intercepts. P - values were calculated using Satterwhaite's method.*

2. Supplementary Figures

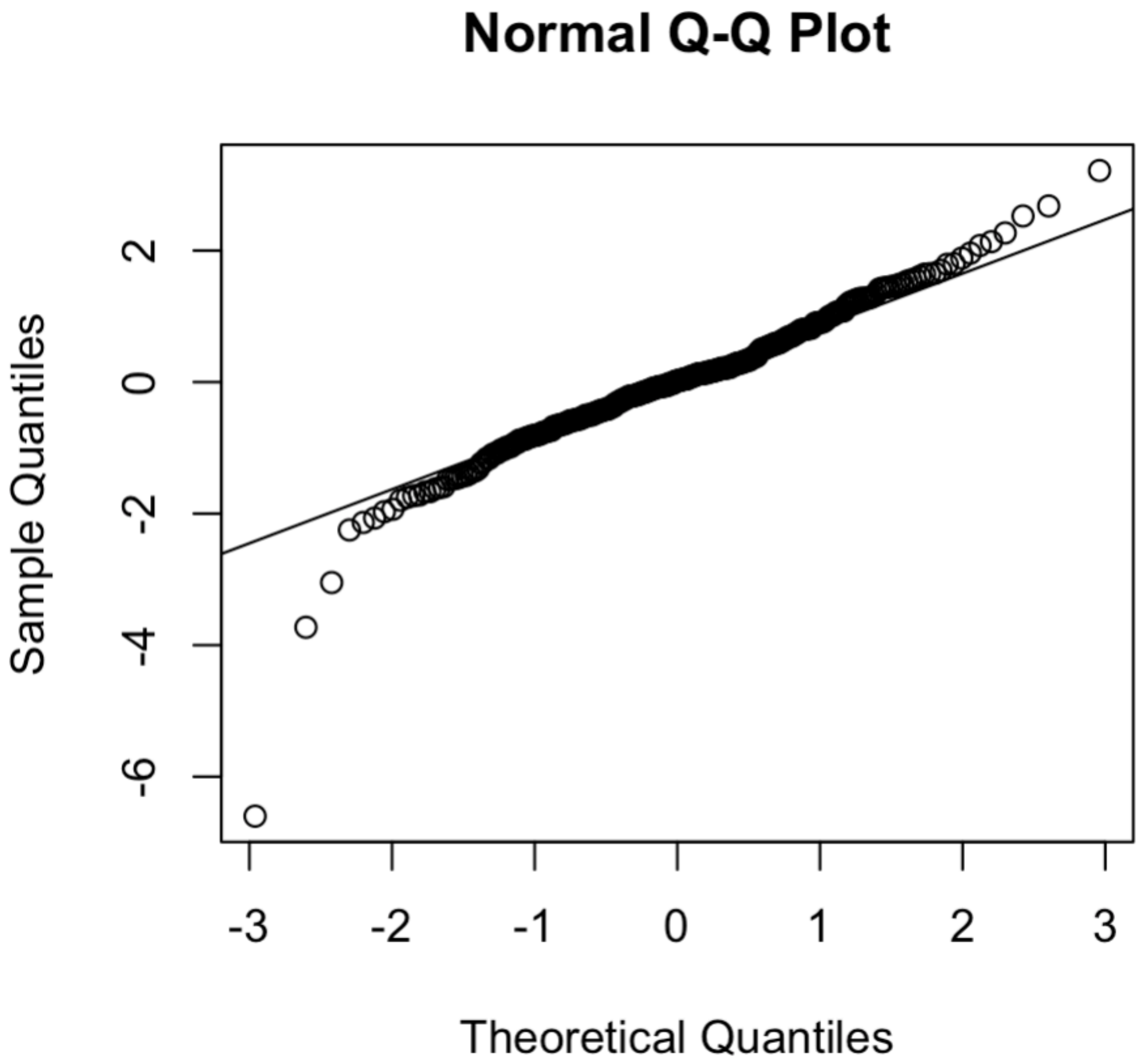

Figure S1. *QQ-plot of standardized residuals of linear mixed effects model shown in Table 3. For sensitivity analysis three outliers on the bottom with standardized residual values of < -3 and one outlier at the top with standardized residual value > 3 was removed*
